# Lactate as Potential Mediators for Exercise-Induced Positive Effects on Neuroplasticity and Cerebrovascular Plasticity

**DOI:** 10.3389/fphys.2021.656455

**Published:** 2021-07-05

**Authors:** Zhihai Huang, Yulan Zhang, Ruixue Zhou, Luodan Yang, Hongying Pan

**Affiliations:** Cognitive and Sports Neuroscience Laboratory, National Demonstration Center for Experimental Sports Science Education, College of Physical Education and Sports Science, South China Normal University, Guangzhou, China

**Keywords:** lactate, physical exercise, neuroplasticity, cerebrovascular function, brain

## Abstract

The accumulated evidence from animal and human studies supports that exercise is beneficial to physical health. Exercise can upregulate various neurotrophic factors, activate neuroplasticity, and play a positive role in improving and enhancing cerebrovascular function. Due to its economy, convenience, and ability to prevent or ameliorate various aging-related diseases, exercise, a healthy lifestyle, is increasingly popularized by people. However, the mechanism by which exercise performs this function and how it is transmitted from muscles to the brain remains incompletely understood. Here, we review the beneficial effects of exercise with different intensities on the brain with a focus on the positive effects of lactate on neuroplasticity and cerebrovascular plasticity. Based on these recent studies, we propose that lactate, a waste previously misunderstood as a by-product of glycolysis in the past, may be a key signal molecule that regulates the beneficial adaptation of the brain caused by exercise. Importantly, we speculate that a central protective mechanism may underlie the cognitive benefits induced by exercise.

## Introduction

Physical inactivity is the fourth leading cause of death worldwide, and it is also a major cause of non-communicable diseases worldwide (Kohl et al., 2012). Strong evidence shows that physical inactivity increases the risk of many adverse health conditions, including coronary heart disease, type 2 diabetes, and breast and colon cancer, and leads to shortens life expectancy (Lee et al., 2012). Not only that, studies have reported that 6–10% of all non-communicable disease deaths worldwide are attributable to physical inactivity, and some diseases are even higher (Lee et al., 2012). Therefore, this presents a significant public health problem worthy of attention.

Growing evidence has shown that physical exercise is beneficial to health, including the brain. In the general population, exercise can improve attention, processing speed, memory, and executive function (Cassilhas et al., 2016; Wu et al., 2018; Hu et al., 2020; Stillman et al., 2020; Sun et al., 2020; Wu et al., 2020). Apart from these, numerous studies have demonstrated that different exercise regimes can significantly improve various brain functions, including regulating cerebrovascular function, enhancing neuroplasticity, inhibiting neuroinflammation, and preventing neurodegenerative diseases (Hotting and Roder, 2013; Ryan and Nolan, 2016; Liu et al., 2019; Malkiewicz et al., 2019; Guadagni et al., 2020). In recent years, the protective effects of physical exercise on neurodegenerative diseases and cerebrovascular diseases have attracted many researchers’ attention. Besides, lactate, a substance that was considered a metabolic waste for a long time in the past, has gradually been proved to play an essential role in exercise-mediated benefits in brain functions.

Since lactate was first discovered in 1780, scholars’ views on it have been continuously changing. For a long time, most researchers believed that lactate was a useless glycolysis product and was the leading cause of muscle fatigue during exercise. However, in the past few decades, new evidence has shown that lactate is an important energy source and plays many critical roles in the body. For instance, astrocyte-neuron lactate transport is necessary for long-term memory formation (Suzuki et al., 2011). Besides, lactate can also ameliorate depression symptoms, potentiate angiogenesis and neurogenesis, and may involve in exercise-induced cerebrovascular changes and the release of brain-derived neurotrophic factor (BDNF; Morland et al., 2017; Zhou et al., 2018; El Hayek et al., 2019; Karnib et al., 2019).

This review aims to elucidate the relationship between lactate- and exercise-induced benefits in brain functions. We first provide recent evidence of exercise-induced positive effects on the brain and further emphasize lactate’s potential to protect the brain and treat brain injury. Finally, we discuss the possible role of lactate in exercise-induced neuroplasticity and cerebrovascular plasticity and highlight the gaps in current research. Understanding these may shed light on clarifying the mechanism by which exercise improves brain function and the possibility of clinical application of lactate, especially for those who cannot enjoy the benefits of exercise due to physical reasons.

## Main Text

### The Beneficial Effects of Exercise on the Brain

Physical activity is defined as any body movement produced by skeletal muscles that require energy expenditure. Physical activity in daily life can be categorized into sports, occupational, household, conditioning, or other activities (Caspersen et al., 1985). Exercise is different from physical activity: It is a subset of physical activity that refers to a planned, organized, repetitive, and purposeful physical activity; it is ultimate, or intermediate purpose is to improve or maintain the adaptability of physical exercise (Caspersen et al., 1985). Although almost all types of appropriate exercise have been shown to be beneficial to brain function and mental health, aerobic exercise has attracted widespread attention due to its beneficial effects on hemodynamics and neuroplasticity. In this section, we will review how exercise regulates neuroplasticity and cerebrovascular function and discuss whether there are differences in physiological benefits brought by different types of exercise.

#### Exercise, Neuroplasticity, and Cerebrovascular Plasticity

“Neuroplasticity” refers to the capacity of the nervous system to adapt and optimize its limited resources in response to physiological changes, injuries, new environmental demands, and sensory experiences (Pascual-Leone et al., 2005). It has long been believed that in the early stages of an individual’s development, the nervous system has tremendous potential for inherent plasticity that gradually fades over time. However, in recent decades, convincing evidence indicates that stimulation, such as environmental changes, physical exercise, or cognitive training, reactivates the adult neocortex’s plasticity, alters cortical circuit activity, and changes the structure or function of the brain, suggesting that brain plasticity may be lifelong (Raz and Lindenberger, 2013; Hubener and Bonhoeffer, 2014; Moreno-Jimenez et al., 2019). It should be emphasized that exercise, a cost-effective method, has great potential in activating neuroplasticity. Specifically, exercise can induce a cascade of molecular and cellular processes that support brain plasticity, thus contributing to cognitive function and resisting the brain function decline caused by aging or nervous system diseases (Knaepen et al., 2010; Hotting and Roder, 2013; Cassilhas et al., 2016; Fernandes et al., 2017). It is also noteworthy that exercise could improve neuroplasticity and positively affects cerebrovascular function (Tan et al., 2014; Nishijima et al., 2016; Dao et al., 2018; Joris et al., 2018).

The human brain occupies 2% of the body mass but consumes 20% of the body’s energy at rest (Andreone et al., 2015). So, it is highly dependent on the oxygen and glucose supply in the blood, which needs to be achieved through an extensive, well-regulated vascular network. Blood–brain barrier (BBB) is a complex structure located between the brain parenchyma and the vascular system, and it can regulate the inflow and outflow of various soluble compounds between the systemic circulation and the brain and restrict the entry of inflammatory cells and other substances into the central nervous system (Zhao et al., 2015). The BBB is composed of vascular cells, glial cells, and neurons, the interaction between these components led to the concept of the neurovascular unit (NVU), and actually, NVU plays an indispensable role in regulating cerebral blood flow (CBF) and maintaining the integrity of the BBB (Iadecola, 2013; Zhao et al., 2015; Sweeney et al., 2018).

Notably, the destruction of NVU may induce chronic vascular insufficiency and hypoperfusion and is also associated with a variety of neurodegenerative diseases (Cai et al., 2017; Kisler et al., 2017; Zenaro et al., 2017; Sweeney et al., 2018). For instance, an early study showed that subjects with a higher CBF (middle cerebral artery on both sides) speed are less likely to develop dementia and have significantly reduced cognitive decline during the same period, while low CBF is associated with a higher prevalence of dementia and related to the markers of early dementia (Ruitenberg et al., 2005). In addition, a cross-sectional study investigated the relationship between the severity of vascular disease and Alzheimer’s disease dementia (Arvanitakis et al., 2016). The results showed that in 1,143 cases, 39% of patients had moderate to severe arteries atherosclerosis, atherosclerosis is present in 35%, every time the severity of atherosclerosis or arteriosclerosis increases, and the probability of suffering from Alzheimer’s disease dementia increases significantly (appears as a lower overall cognitive score; Arvanitakis et al., 2016). Collectively, these studies emphasize the importance of maintaining normal cerebrovascular function.

On the other hand, the cerebrovasculature is also plastic. Under normal circumstances, the cerebrovascular structure is largely static. However, when the brain microenvironment changes due to disease, the external environment, or trauma, the body promotes angiogenesis and vasculogenesis by increasing key factors, such as vascular endothelial growth factor (VEGF) to maintain a stable state (Fabel et al., 2003; Bogorad et al., 2019). Therefore, cerebrovascular plasticity is a process that causes changes in the architecture of brain microvessels (Bogorad et al., 2019). Accumulating evidence shows that moderate exercise under normal or pathological conditions can effectively promote angiogenesis-related factor production, improve cerebrovascular plasticity, and optimize cerebrovascular function (Nishijima et al., 2016; Dao et al., 2018; Kwak et al., 2018; Xie et al., 2019). Importantly, exercise provides a variety of health besides hemodynamics compared to other traditional interventions and may be more conducive to maintain or repair NVU function. For many cerebrovascular diseases, exercise undoubtedly offers encouraging hope.

As mentioned above, it has been widely recognized that exercise can bring many positive effects on the brain. When summing up these works in most studies, however, it is easy to overlook that different types of exercise may cause different physiological effects, which may make some conclusions imprecise. Therefore, in the following sections, we will discuss the benefits of different kinds of exercise on brain function.

#### Benefits of Moderate- and Low-Intensity Exercise on Neuroplasticity and Cerebrovascular Plasticity

For many reasons, the past guidelines often recommended people to carry out moderate- and low-intensity exercise to maintain health (MLIE). MLIE is also the most commonly used paradigm in sports medicine research. Indeed, accumulating evidence supports the beneficial effects of MLIE on neuroplasticity and cerebrovascular plasticity. A randomized controlled trial was conducted in 120 elderly adults showed that aerobic exercise could increase the size of the anterior hippocampus. In this study, subjects were divided into an aerobic exercise group (50–75% maximum heart rate reserve) and stretching exercise group (corresponding to the appropriate intensity to reach 13–15 points on the Borg self-induced fatigue scale). After a one-year intervention, the left and right hippocampal volumes in the aerobic exercise group increased by 2.12 and 1.97%, respectively, while Brain-derived neurotrophic factor (BDNF) increased significantly, accompanied by improvement in memory function; however, the hippocampal volume in the stretching control group decreased by 1.40 and 1.43%, respectively (Erickson et al., 2011). Notably, the hippocampus is a vital region for learning and memory. Its atrophy may be closely related to cognitive decline (Zheng et al., 2018). Given that hippocampal volume normally decreases by 1–2% per year in the elderly, moderate intensity of aerobic exercise can effectively reverse the age-related loss of hippocampal volume by 1–2 years, while lower intensity of stretching exercise may be insufficient to counteract age-related hippocampal atrophy (Raz et al., 2005; Erickson et al., 2011). Similarly, for healthy adults (19–65 years old), balanced exercise training lasting 50 min twice a week for a total of 12 weeks can activate the neuroplasticity of the brain area related to vision and vestibular self-motor perception, which is reflected in increased cortical thickness in the superior temporal cortex, visual association cortices, the posterior cingulate cortex, the superior frontal sulcus, and the precentral gyri (Rogge et al., 2018).

Evidence from animal studies also supports that exercise can enhance brain plasticity. Moderate-intensity aerobic exercise promotes the proliferation and survival of neurons in the hippocampus and cortex, increased BDNF and dendrites, and improves spatial learning and memory in rodents (Serra et al., 2019; Ivy et al., 2020). Another study also reported that moderate-intensity swimming training could ameliorate the anxiety-like behavior of mice by increasing proteins that play a key role in hippocampal plasticity, such as growth-associated protein 43, synaptophysin, and BDNF (Liu et al., 2018).

Similarly, MLIE also showed many positive effects on cerebrovascular plasticity. In an animal model, low-intensity aerobic exercise after stroke can promote angiogenesis in the lesion area, increase microvessel density, and improve functional outcomes (Zhang et al., 2013). Other related studies further support these results (Ma et al., 2013; Cahill et al., 2017; Hong et al., 2020; Leardini-Tristao et al., 2020). Clinically, aerobic exercise with 70% HRmax intensity twice a week for 12 weeks increased the diameter of cerebral microvasculature in migraine patients, promoted their cerebrovascular health, and effectively reduced the number of days they reported migraine (Hanssen et al., 2018). Furthermore, available evidence indicates that exercise could increase CBF to a certain extent (van der Kleij et al., 2018; Kleinloog et al., 2019). Unfortunately, there is no research to confirm the relationship between improved CBF and changes in the brain’s microvascular structure.

Taken together, both animal and human studies reported the positive effects of MLIE on neuroplasticity and cerebrovascular plasticity. Nonetheless, the specific mechanisms of this effect caused by exercise are still not fully understood. Understanding the mechanism of exercise-induced plasticity will help promote exercise rehabilitation in the fields of mental health, neurodegenerative diseases, and acquired brain injury.

#### Can High-Intensity Exercise Produce Equal Effects?

High-intensity exercise may seem equivalent to excessive exercise or exhaustive exercise. Also, the traditional exercise strategies recommended by relevant guidelines are often based on moderate-intensity exercise, so we instinctively rarely think about the benefits of high-intensity exercise. Interestingly, recent and accumulating work in animal studies and clinical trials indicates that high-intensity exercise, especially high-intensity interval training (HIIT), could produce physiological benefits similar to or higher than those of conventional continuous MLIE (Lezi et al., 2014; Wahl et al., 2014; Lucas et al., 2015; Stensvold et al., 2020; Hu et al., 2021). It is worth noting that high-intensity exercise-induced brain plasticity may also be more beneficial than MLIE.

Changes in the strength of synaptic communication in the cerebral cortex can reflect its plasticity, which some forms of noninvasive brain stimulation can assess. In the study of Andrews et al., transcranial magnetic stimulation was used to evaluate the effects of HIIT (50% HR max, 4 × 3 min and 90% HR max, 4 × 2 min) and moderate-intensity continuous exercise (50%HRmax, 20 min) on the plasticity of the motor cortex. The results show that HIIT can more effectively stimulate the plasticity of the motor cortex, as evidenced by increased excitability and decreased inhibition of the cortex. In contrast, the effect of moderate-intensity exercise is somewhere between HIIT and resting (Andrews et al., 2020).

Recently, a notable study compared cerebral hemodynamics and circulating neurotrophic factor responses to three different types of exercise regimens in healthy adults. They found an interesting phenomenon, compared with the traditional continuous exercise (65% VO2peak, lasting 30 min) and HIIT (85% HRmax, 4 × 4 min), and ultra-high-intensity intermittent exercise with 200% maximum aerobic capacity (4 × 30 s) could maximize the increase of VEGF and BDNF (Weaver et al., 2020). More importantly, the lactate produced by this training paradigm showed the most significant increase, suggesting the potential connection of lactate with these factors. Indeed, growing evidence has shown that high-intensity exercise can increase the level of BDNF, a critical nutritional factor promoting neuroplasticity, to a greater extent than MLIE (Jimenez-Maldonado et al., 2018; Rodriguez et al., 2018; Cefis et al., 2019). Other animal studies also highlight the positive effects of high-intensity exercise on the brain (Saucedo Marquez et al., 2015; Dos Santos et al., 2020; Hu et al., 2021).

As a more time-saving and practical method to optimize health, HIIT has been widely investigated and promoted in recent years. But undeniably, any under-planned exercise comes with certain risks, especially high-intensity exercise. In many cases, it will be accompanied by the increase of corticosterone, inflammatory factors, reactive oxygen species, or other stimulating factors, which often have adverse effects on brain function (Rosa et al., 2007; Powers et al., 2016; Sun et al., 2017; Radak and Powers, 2020). Due to differences in research paradigms, exercise programs, and subject groups, the extent to which high-intensity exercise is beneficial or harmful has not yet been determined, and many studies come to contradictory conclusions. Future research should focus on obtaining the benefits of high-intensity exercise while minimizing its possible detrimental effects and using more appropriate evaluation tools to select a reasonable exercise program for individuals.

### Lactate: Past and Future Directions

#### Past and Present Views on Lactate

In 1780, the Swedish chemist Carl Wilhelm Scheele first discovered lactate in sour milk; this substance was named “Mjölksyra,” which means “acid in milk” (Ferguson et al., 2018). Since then, lactate has become the focus of discussion and research by scientists, and with the deepening of research, we continue to have new understandings about lactate. For a long, the theory that lactate is a glycolytic waste product has been dominant, and muscle research at that time believed that lactate was produced by the body through anaerobic glycolysis, which directly leads to metabolic acidosis, damages muscle damage, and causes muscle fatigue and soreness (Hall et al., 2016; Magistretti and Allaman, 2018). So it is also called “lactic acid,” but actually, this term is controversial. Subsequent studies clarified that the root cause of muscle soreness after exercise is caused by delayed onset muscle soreness rather than lactate, and in the 1930s, studies pointed out that excessive lactate can be oxidized to produce pyruvate in specific organs, which enters the tricarboxylic acid cycle and produces glucose through gluconeogenesis (Magistretti and Allaman, 2018; Rubin, 2019). Unfortunately, this lactate cycle was simply considered a way for the body to eliminate this harmful metabolite.

Until Schurr et al. (1988) discovered that lactate could support normal synaptic function. They first exposed rat hippocampal slices to the standard artificial cerebrospinal fluid containing glucose, and the synaptic function showed active, and when the perfusion medium is replaced with a medium without glucose, the synaptic function gradually weakens, then they were progressively perfused with lactate in the culture medium without glucose, and the synaptic function of hippocampal slices increased in a dose-dependent manner, completely replacing the function of glucose, which indicates that *in vitro* lactate can be used as the sole substrate of energy metabolism to support synaptic function (Schurr et al., 1988). Since then, researchers have begun to re-examine the role of lactate in the body, and the hypothesis of the astrocyte-neuron lactate shuttle further verifies that lactate can be transported to neurons *via* monocarboxylate transport and used by neurons as an energy source (Pellerin and Magistretti, 1994; Pellerin et al., 1998; Bergersen, 2007). Subsequently, Gallagher and collaborators (Gallagher et al., 2009) proved that the human brain could use lactate as an energy source for the first time. More studies have also shown that astrocyte-neuronal lactate transport plays a critical role in the formation and maintenance of long-term memory, indicating lactate may be a signaling molecule in the brain (Pellerin et al., 1998; Bergersen, 2007). Notably, in both animal and clinical studies, there is continuous evidence that lactate has neuroprotective and therapeutic effects on different types of encephalopathy, even in some psychiatric disorders (Berthet et al., 2009, 2012; Alvarez et al., 2014; Carrard et al., 2018; Zhou et al., 2018; Karnib et al., 2019).

Overall, from the waste product of glycolysis to important energy substrates and signaling molecules in the body, these studies emphasized that the role of lactate is much more important than we thought in the past. Lactate, a mysterious substance, still needs continuous exploration to understand its role in the body.

#### Lactate Metabolism in the Brain

In general, there are two main ways to produce lactate in the brain, namely blood lactate circulation and the glycolysis of glycogen in astrocytes. The transport of blood lactate is accomplished by the monocarboxylic acid transporter (MCT). Among the identified MCTs, only MCT1 to MCT4 can transport monocarboxylates through the cell membrane, including lactate, pyruvate, and ketone bodies (Halestrap and Price, 1999). During exercise, when the working muscle contracts to a certain intensity (usually we express this critical point as the lactate threshold), the muscle will produce lactate and release it into the blood, while a portion of blood lactate can permeate through the BBB under the action of MCT and enter the brain (Bergersen, 2015).

The accumulation of lactate in the brain was once considered the reason for central fatigue caused by exercise. However, studies based on lactate kinetic evaluation confirmed that blood lactate circulating to the brain is its main energy substrate during exercise, which is evidenced by the increase of lactate utilization rate and the decrease of glucose utilization rate (Kemppainen et al., 2005; Quistorff et al., 2008). Correspondingly, astrocyte-derived lactate is also essential for the maintenance of many important brain functions. Typically, glucose, which enters neurons and astrocytes through the BBB, is an essential source of energy for the brain, and astrocytes will store a portion of this as glycogen (Pellerin et al., 1998; Suzuki et al., 2011; Proia et al., 2016). When the body is in a period of aglycemia and increased metabolic demand, neuronal activity will make the glycogen stored in astrocytes rapidly degrade into lactate. This disperses in the extracellular space through MCTs, providing an energy source for neurons to maintain neuronal function (O’Dowd et al., 1994; Brown et al., 2004). Although significant progress has been made in related fields in recent years, many mysteries remain in this astrocyte-neuron lactate transport system. Why does the brain preferentially mobilize astrocyte-derived lactate as an energy substrate under these conditions? Are the effects and mechanisms of lactate in two pathways different on the brain? To address this knowledge gap, a great deal of research is required. Preliminary studies indicated that exogenous injection of lactate could rapidly increase lactate signals in astrocytes and neurons, suggesting there may exist a coordinated effect (Machler et al., 2016).

#### The Effects of Lactate on the Brain

For astrocyte-derived lactate, Suzuki’s research team has made significant findings before (Suzuki et al., 2011). They reported that during learning tasks in rats, hippocampal astrocytes released a large amount of lactate into extracellular space, accompanied by a significant increase in the expression of MCT1. Importantly, this was necessary to maintain long-term potentiation (LTP), a neuronal signal enhancement phenomenon that constitutes the basis of learning and memory; Blocking glycogen metabolism in astrocytes could destroy the maintenance of LTP but not prevent its induction, while blocking MCT4, MCT1, and MCT2 completely eliminated the LTP effect, and rats showed amnesia. Interestingly, injection of L-lactate into the bilateral hippocampus rescued this LTP destruction but had no effect on the LTP decreases caused by blocking MCT2 alone (Suzuki et al., 2011). Although many phenomena cannot be fully explained yet, they speculate that the learning triggers a series of events in the brain that lead to short-term and long-term memory. In contrast, long-term memory formation is accompanied by the activation of neural networks and higher metabolic demands, thus causing astrocytes to release lactate to supply active neurons. In addition, astrocyte-derived lactate is also crucial for maintaining brain function during exhaustive exercise. Exhaustive exercise can greatly reduce glycogen levels in muscles and the hippocampus, and elevate MCT2 and lactate in the brain. Meanwhile, blocking astrocytic glycogen decomposition or MCT2 can significantly reduce lactate signals and ATP levels in the brain, resulting in brain fatigue (Matsui et al., 2017). Following experimental cerebral ischemia in rats, lactate was observed to accumulate around the hematoma and enhance neurogenesis and angiogenesis, which could be prevented by inhibiting endogenous lactate, resulting in serious brain injury and neurological deficits (Zhou et al., 2018). Furthermore, there was a marked increase in lactate content during choroidal neovascularization, which may also be the result of astrocytes responding to changing energy metabolism needs (Song et al., 2018).

Exogenous administration of lactate also yields many exciting findings. In animals, both *in vivo* and *in vitro* experiments have shown that injection of L-lactate after cerebral ischemia resulted in a neuroprotective effect, reduced the degree of tissue damage, and improved the outcomes of the nervous system (Berthet et al., 2009, 2012). Notably, the study also observed excessive L-lactate injection could also exert toxic effects on hippocampal slices. Indeed, lactate therapy can also effectively improve the outcomes of traumatic brain injury (TBI) patients. A clinical trial conducted by Bouzat and his colleagues (Bouzat et al., 2014) involved the intravenous infusion of hypertonic sodium lactate in patients with severe TBI. They found that early infusion of lactate after TBI improved the brain energy metabolism of the patient by increasing the utilization of extracellular pyruvic acid and glucose, which was also related to the reduction of intracranial pressure and brain glutamic acid (Bouzat et al., 2014). Furthermore, a study reported that lactate-releasing implantable biomaterial scaffold could induce brain injury mice to produce neurons and glial cells continuously and promote angiogenesis of the implants. These new neurons survived for more than a year, differentiated in scaffolds, received synapses and sent axons, and integrated into functional brain circuits (Alvarez et al., 2014). Exogenous lactate administration has also been shown in other animal studies to promote neuroplasticity (Yang et al., 2014; Lev-Vachnish et al., 2019).

Interestingly, lactate may also be a potential novel antidepressant. In different animal models of depression, peripheral administration of L-lactate, especially long-term administration, could reduce depression-like behaviors similarly to antidepressants, thus partially restoring social avoidance behavior (Carrard et al., 2018; Karnib et al., 2019). However, some studies have different conclusions about the impact of lactate. Data from Hayek’s work (El Hayek et al., 2019) show that exogenous lactate injection can also improve mice’s cognitive learning and memory. On the contrary, Lev (Lev-Vachnish et al., 2019) reported that long-term injection of lactate could promote nerve regeneration, but observed no improvement in animals’ cognitive learning and memory ability. One possible reason is that long-term and short-term injections of lactate and the choice of dosage will have different effects on animals. Early research has shown that lactate injection is related to the onset of acute anxiety in susceptible individuals (Pitts and McClure, 1967).

Overall, these studies show that lactate exhibits pleiotropic effects in the brain. A better understanding of the molecular mechanism of lactate action will also open the way to develop targeted treatments.

### What Mediates Exercise-Induced Brain Plasticity: The Possible Involvement of Lactate

Most researchers believe that a possible mechanism of exercise-induced brain plasticity is related to the increased expression or downstream signaling of neurotrophic factors, such as BDNF, VEGF, and insulin-like growth factor 1 (IGF-1). Actually, in rodents, blocking the signal conduction of BDNF in the hippocampus can attenuate exercise-induced neuroplasticity (Vaynman et al., 2004; Griesbach et al., 2009), and blocking the entrance of circulating IGF-1 or VEGF into the brain can inhibit hippocampal neurogenesis caused by exercise (Trejo et al., 2001; Fabel et al., 2003). Notably, IGF-1, a wide-spectrum growth factor with angiogenic actions, is also essential for exercise-induced cerebral angiogenesis. Studies have shown that systemic injection of IGF-1 can stimulate angiogenesis, and inhibiting its effect can reduce the increase in cerebral blood vessel density induced by exercise (Lopez-Lopez et al., 2004).

Of note, irisin/fibronectin type III domain-containing protein 5 (FNDC5), a hormone-like myokine, is also valued by researchers because of its regulatory role in the beneficial effects of exercise (Zhang and Zhang, 2016; Islam et al., 2017; Lourenco et al., 2019; Young et al., 2019). Irisin is a cleaved form of FNDC5, while FNDC5 is a transmembrane precursor protein under the control of PGC-1α (Islam et al., 2017). The expression of FNDC5 gene in skeletal muscle increases in response to exercise, leading to increased circulating irisin and brings a series of beneficial effects, and the PGC-1α/FNDC5/BDNF pathway has been regarded as an important neuroprotective pathway in recent years. In 2012, when Bostrom’s research group discovered this new secreted form of FNDC5, they established that FNDC5 is a PGC-1α-dependent myokine, which is secreted from muscles during exercise and regulates the main body metabolic benefits induced by exercise, such as fat cell browning and improvement of glucose homeostasis (Bostrom et al., 2012), other studies further prove these crucial roles of irisin (Huh et al., 2014; Lee et al., 2014; Perakakis et al., 2017). In addition to muscles, irisin/FNDC5 is profoundly expressed in many areas of the brain, including Purkinje cells in the cerebellum, hypothalamus, and hippocampus (Dun et al., 2013; Wrann et al., 2013; Varela-Rodriguez et al., 2016), and is involved in the regulation of some vital brain functions (Zhang and Zhang, 2016). For instance, in the process of neural differentiation of mouse embryonic stem cells, the presence of FNDC5 plays an indispensable role. Knockout of FNDC5, whether during or after the formation of neural progenitor cells, will result in significantly decreased levels of mature neuron markers (Forouzanfar et al., 2015). During the differentiation of neural progenitor cells derived from human embryonic stem cells into neurons, high expression of FNDC5 was also observed (Ghahrizjani et al., 2015). Also, one study reported that exercise could induce the production of irisin in the hippocampus and increase the expression of BDNF; furthermore, the delivery of FNDC5 to the liver by peripheral injection can also lead to an increase in blood irisin, thereby inducing the expression of hippocampal BDNF and other neuroprotective genes (Wrann et al., 2013). Emerging evidence has indicated that irisin is an essential mediator of the neuroprotective effects of exercise. A study initiated by Lourenco and collaborators found that the level of irisin is significantly reduced in the hippocampal slices of patients with Alzheimer’s disease, and in AD animal models, blocking Irisin/FNDC5 can weaken the protective effect of exercise on synaptic plasticity and memory impairment caused by AD; interestingly, increasing the level of irisin/FNDC5 in the brain can rescue this defect in AD mice (Lourenco et al., 2019). Similarly, in a mouse model of cerebral ischemia, the blockade of irisin/FNDC5 can also weaken the protective effect of exercise on neurological deficits caused by cerebral ischemia; compared with the control group, the blocking of irisin/FNDC5 performed more serious neuronal damage (Li et al., 2017).

The discovery of irisin certainly provides new possibilities for treating many diseases, but the mystery of irisin has not yet been fully revealed. Whether peripheral irisin/FNDC5 can reach the brain or trigger the increase of irisin/FNDC5 in the brain through a certain mechanism is still a hot topic of debate. Also, little is known about the initial molecular signals of exercise-induced neuroplasticity and how they are transmitted from muscles to the brain, and potential upstream or downstream mechanisms in the PGC-1α/FNDC5/BDNF pathway are yet to be confirmed by prospective studies. Here, we show several recent studies. Data from these studies indicate that lactate or its receptors are involved in various effects of exercise on the brain and may be the initial molecular signal between exercise and beneficial adaptation of the brain.

Practically, previous studies have found that exogenously administered lactate can reproduce part of the body’s adaptive changes induced by exercise (approximate the lactate threshold intensity, 7 weeks in a row), including liver and brain (Lezi et al., 2013). To the best of our knowledge, the study conducted by Morland et al. (2017) proved for the first time the connection between exercise, lactate, and cerebrovascular remodeling. They found that both HIIT (90% maximum oxygen uptake) for 5 days per week for 7 weeks and subcutaneous injection of L-lactate can increase blood lactate levels, brain VEGF, and capillary density in mice (Morland et al., 2017). Notably, a high concentration of the lactate receptor, hydroxycarboxylic acid receptor 1 (HCAR1), also known as HCA1 or GPR81, was observed in perivascular leptomeningeal fibroblast-like cells and periintracerebral microvasculature cell-like cells feeding the brain, whereas these changes were not observed at all in HCAR1 knockout mice.

Furthermore, a study by El Hayek et al. (2019) showed that lactate could activate the PGC-1α/FNDC5/BDNF pathway in a silent information regulator 1 (SIRT1)-dependent manner (El Hayek et al., 2019). Their research demonstrated that after 30 days of voluntary exercise or a single intraperitoneal injection of lactate increased lactate levels in the mouse brain, which induced hippocampal BDNF expression and TrkB signal transduction and was related to the improvement of spatial learning and memory in mice. In addition, both exercise and lactate injection can increase the activity of SIRT1 in the hippocampus, and the *in vitro* culture results further prove that the expression of lactate-mediated PGC1-α and BDNF is SIRT1 dependent (El Hayek et al., 2019). These results emphasized the possible link between lactate and irisin signal transduction.

However, there are some limitations in this research. They adopted the model of voluntary exercise but did not monitor and evaluate the activity of animals. It is difficult for the voluntary exercise paradigm to reach the threshold strength of lactate that makes muscles release lactate into the blood. Instead, they only tested the lactate level in mice’s brains, without monitoring the lactate levels in the muscles. Therefore, they arbitrarily believe that the lactate released by muscles can pass through BBB and enter the brain to cause a series of effects. This is a problem worthy of attention. We speculate that the increase in lactate in the brain they observed was released by astrocytes. More importantly, does this further suggest that even weak exercise can cause astrocyte-neuron lactate transport?

Furthermore, a follow-up study by Morland and colleagues (Lambertus et al., 2020) demonstrated that exercise had a strong HCAR1-dependent effect on adult neurogenesis in the subventricular zone (SVZ). Using the same HIIT paradigm or injection of L-lactate could activate HCAR1 and promote neurogenesis in SVZ. Still, it was ineffective in HCAR1 knockout mice, indicating that lactate-activated HCAR1 induced neurogenesis in the SVZ. Several studies in humans also point to the link between lactate and BDNF. An early study in male sport students indicated intravenous injection of 4.2 mmol of lactate at rest could increase the expression of BDNF in the blood (Schiffer et al., 2011). Another study reported that compared with resting control, subjects after acute sprint interval exercise have significantly higher levels of peripheral blood lactate and are associated with higher levels of BDNF, IGF-1, and VEGF, which is also related to better performance in cognitive function tests (Kujach et al., 2019). A recent study also highlighted the potential link between lactate and neuroplasticity (Weaver et al., 2020).

**Figure 1:**
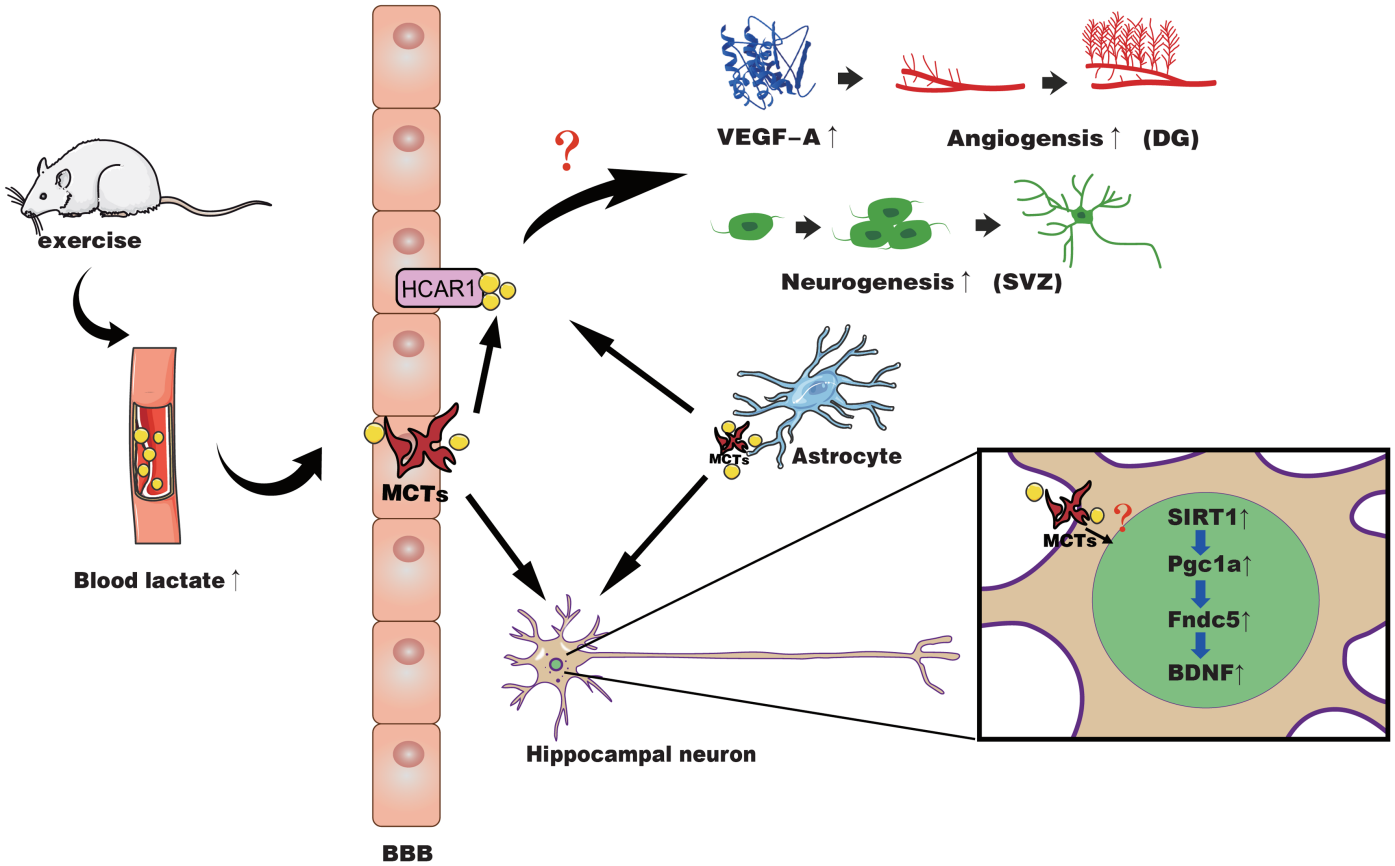
The possible mechanisms of lactate in exercise-induced positive effects on neuroplasticity and cerebrovascular plasticity. Exercise causes muscles to produce lactate, which is then released into the blood and passes through the BBB through different MCTs into the brain parenchyma. Meanwhile, astrocytes may also release lactate during exercise to supply the neural network in response to activated neurons. At the BBB of the microvasculature, lactate binds to HCAR1 to activate it and induces increased VEGF-A expression in the hippocampal dentate gyrus (DG), angiogenesis, and neurogenesis of SVZ through an unidentified pathway. In hippocampal neurons, lactate is transported across the membrane by MCT, activating the SIRT1/PGC1-α/FNDC5/BDNF pathway through an unknown pathway, thereby improving neural plasticity.

In summary, these studies indicate lactate may be a molecular signal used by specific neurotrophic factors *via* certain mechanisms to activate neurogenesis or cerebrovascular plasticity. The potential mechanisms of lactate and exercise-induced effects on the brain are shown in Figure 1. There is also controversy about the way lactate affects changes in the brain. Some research results indicate that lactate can activate the PGC1-α/FNDC5/BDNF pathway through SIRT1 or act through NMDA, while some results show it is HCAR1 (Yang et al., 2014; Morland et al., 2017; El Hayek et al., 2019; Lambertus et al., 2020). Even studies reported that lactate promotes choroidal neovascularization by upregulating VEGF expression in macrophages, regardless of the activity of lactate transporter MCT (Song et al., 2018). In the following section, we will further discuss the gaps in current research.

### Controversies and Gaps in Current Research

Although mounting evidence connects lactate to exercise-induced neuroplasticity and vascular plasticity, unfortunately, the current research fails to separately explore the roles of two pathways of lactate (blood lactate and astrocyte-derived lactate). The critical role of astrocyte-derived lactate on exercise’s effects on the brain is reflected in the work of Soya et al. (Okamoto et al., 2012; Matsui et al., 2017). Here, we present the controversies and gaps in the current work and our hypotheses based on the existing evidence.

First, we investigate the “lactate paradox.” It is well known that blood lactate levels increase significantly only when exercise intensity exceeds the lactate threshold. Morland et al. show that lactate produced by high-intensity exercise mediates the beneficial effects of exercise on the brain (Morland et al., 2017; Lambertus et al., 2020). Paradoxically, moderate- and low-intensity exercise that does not increase blood lactate has many beneficial effects on the brain in many studies, including improvements in BDNF levels, nerve regeneration, vascular regeneration, and cognitive function. In fact, different types of exercise can activate brain neuron activity (Matsui et al., 2011; Hyodo et al., 2012; Soya et al., 2018). We speculate that the activation of neurons in the brain also leads to high metabolic demand during moderate- and low-intensity exercise, thus mobilizing the astrocytes to release lactate to supply the neural network. While high-intensity exercise mobilizes lactate, the blood lactate entering the brain through MCT will cause additional effects, explaining why the high-intensity exercise that produces high lactate can induce greater enhancements in neuroplasticity and cerebrovascular plasticity.

Second, if the above conjecture is true, it will give rise to a lot of questions. For example, will blocking the lactate production pathway of astrocytes in the high-intensity exercise paradigm partially reduce the benefits of exercise to the brain? Would blocking the astrocyte pathway or knocking out HCAR1 during moderate- and low-intensity exercise also reduce or eliminate these benefits? Furthermore, blocking HCAR1 during high-intensity exercise eliminates the benefits of exercise for the brain. Does this mean that HCAR1 and MCT play equally important roles in both lactate-brain pathways?

Thirdly, the previous literature shows that exercise increases the expression of MCTs in multiple brain regions, especially MCT2 (MCT2 is mainly expressed in neurons), which is related to the increase of BDNF and TrkB signals (Takimoto and Hamada, 2014). Similarly, BDNF also promotes MCT2 levels (Robinet and Pellerin, 2010, 2011). Given the vital role of BDNF in maintaining and improving cognitive function and the possible participation of lactate in this mechanism, we suspect that exercise-induced cognitive benefits are likely the result of a central protection mechanism. As noted above, Soya et al. (Matsui et al., 2017) demonstrated that the body would preferentially mobilize astrocytes to release lactate to protect brain function during exhaustive exercise, and the level of lactate was closely related to BDNF (El Hayek et al., 2019; Lev-Vachnish et al., 2019; Muller et al., 2020). These studies provided the foundation for this hypothesis. Therefore, long-term exercise may increase MCT expression in the body, including the brain. The lactate transported to neurons through two pathways is increased, which increases BDNF levels and then promotes the expression of MCT2, forming a beneficial feed-forward loop that maintains long-term cognitive benefits.

Finally, the role of lactate receptor HCAR1 may not be ignored. Although agonist stimulation of HCAR1 alone does not produce a similar effect to lactate (Lev-Vachnish et al., 2019), one study has also shown that HCAR1 signaling supports the process of memory consolidation in the absence of lactate metabolism (Scavuzzo et al., 2020), and HCAR1 is also involved in the regulation of some neural networks (de Castro Abrantes et al., 2019). Besides, there are still few studies on the specific link between lactate and PGC-1α/FNDC5/BDNF pathway, especially the lack of relevant *in vivo* studies. This is still a nascent field, and more research is needed to understand these underlying mechanisms.

## Conclusion

Exercise is beneficial for body and brain health, which has been generally accepted by people. In today’s rapidly aging society, age-related memory decline and neurodegenerative diseases, such as Parkinson’s disease and Alzheimer’s disease, may become more prevalent. In some highly stressed groups, the incidence of depression cannot be ignored. Research has proved that exercise can effectively prevent or combat these diseases, highlighting the importance of exercise. However, the intensity and types of exercise should be considered when starting an exercise program. More importantly, due to physical weakness caused by diseases and aging, not everyone may be able to enjoy the benefits of exercise. Therefore, in recent years, there have been related studies trying to develop a drug that can simulate the benefits of exercise on the body and brain (Guerrieri et al., 2017; Choi et al., 2018; Hawley et al., 2021). In this review, we focused on the role of lactate in exercise-induced neuroplasticity and cerebrovascular plasticity. These studies indicate lactate is likely to be a key signal molecule that regulates beneficial adaptation of the brain caused by exercise. However, how lactate mediates this effect is still unknown. Therefore, further research is needed to determine this effect of lactate and the neurobiological mechanism, especially its possible participation in the PGC1-α/FNDC5/BDNF signaling pathway. The establishment of these molecular pathways will facilitate the application of lactate to clinical treatment. For example, activating some lactate mediation in the brain function may enhance the benefits of physical exercise for the body and brain. Practically, current evidence suggests that HIIT induces higher BDNF levels and beneficial cerebrovascular changes compared to moderate- or high-intensity continuous exercise. Still, some people cannot enjoy the benefits of this higher-intensity exercise due to physical reasons. Suppose lactate therapy can be developed into a pill that partially mimics the benefits of exercise. Such a treatment could help more people to maintain the physical and brain health effects of exercise with a low-cost means.

## Author Contributions

ZH and YZ reviewed the literature and drafted the manuscript. ZH and RZ drew the Figure. LY and HP revised and supervised the project. All authors read and approved the final manuscript.

### Conflict of Interest

The authors declare that the research was conducted in the absence of any commercial or financial relationships that could be construed as a potential conflict of interest.
